# Development of the COMPASS model of endometriosis: A COmprehensive model of pain encompassing agency, systemic factors and sense making

**DOI:** 10.1111/bjhp.12794

**Published:** 2025-03-18

**Authors:** Brydee Pickup, Louise Sharpe, Jemma Todd

**Affiliations:** ^1^ School of Psychology, Faculty of Science University of Sydney Sydney New South Wales Australia

**Keywords:** biopsychosocial model of pain, endometriosis, invalidation, mixed methods, pain

## Abstract

**Objectives:**

Endometriosis is a chronic and progressive condition commonly associated with debilitating pain. Treatments for endometriosis pain are limited and usually invasive. Psychological interventions are a non‐invasive intervention option and have proven benefits in chronic pain. Yet, psychological interventions for endometriosis pain are scant and of limited efficacy, which may be due to gaps in our understanding of endometriosis pain experiences. We sought to expand current understandings of endometriosis pain by investigating the factors that exacerbate and alleviate pain‐related impact and distress, despite similar levels of pain severity.

**Design:**

A mixed‐methods approach was used, comprising quantitative pain data, qualitative interviews, and qualitative analysis of open‐ended survey responses.

**Methods:**

A total of 873 participants answered an online survey including pain outcomes. Sixteen participants were then purposively sampled for interview, in an iterative manner, in line with grounded theory until theoretical saturation was reached. Analysis of interview data resulted in a novel model of endometriosis pain. The model was cross‐validated and refined using content analysis of 841 open‐ended online survey responses regarding wider system priorities for endometriosis care.

**Results:**

Our COMPASS model suggested that experiences of endometriosis‐related pain and associated distress and impact were shaped by a dynamic interaction between the challenges of the gendered nature of pain, invalidation, distrust in the healthcare system, agency, sense‐making, and burden. These experiences were situated within broader systemic factors of difficulty accessing care, the limitations of available treatments, and a lack of financial support.

**Conclusions:**

Our findings present a novel model of endometriosis pain. This model provides several psychosocial treatment targets that could inform future psychological and multidisciplinary interventions for endometriosis pain. Empirical validation of the model is an avenue for future research.

**Statement of contribution**

**What Is Already Known on this Subject?**

- Endometriosis is a chronic, progressive condition associated with debilitating pain.
- Endometriosis‐related pain, distress, and interference do not often correspond to the condition's pathology, suggesting the involvement of psychosocial factors, as in other pain conditions.
- Psychological interventions for endometriosis pain are limited, and there is currently no biopsychosocial model of endometriosis pain to inform the development of new specific and efficacious interventions.

**What Does this Study Add?**

- The first biopsychosocial model of endometriosis pain, cross‐validated by responses from 841 individuals with endometriosis.
- Potential psychosocial treatment targets for alleviating endometriosis‐related pain, distress and interference.

## INTRODUCTION

Endometriosis is a chronic condition that occurs when endometrial‐like cells, commonly found in the uterus, infiltrate other parts of the body and induce inflammation (Dunselman et al., 2014). The condition affects 1 in 9 people born with a uterus (Rowlands et al., 2021) and is associated with a wide symptom profile, including pain, fatigue, sleep and gastrointestinal disruptions, and infertility (Evans et al., 2018). Up to 80% of people with endometriosis report related pain (Bulletti et al., 2010). These pain experiences may comprise any combination of pelvic pain (which may be constant, fluctuating or with unpredictable flares), dysmenorrhea, dyspareunia, and dyschezia (Giudice & Kao, 2004). Individuals with endometriosis‐related pain experience worse quality of life and mental health outcomes compared to individuals without endometriosis‐related pain (Facchin et al., 2015; Gambadauro et al., 2019). Pain is therefore a particularly impactful symptom.

Endometriosis‐related pain has been predominantly medically managed, including surgeries to excise endometriosis lesions and hormonal therapies to prevent further growth. However, these approaches are costly to the individual and medical systems and have limited efficacy (Becker et al., 2017). One in four individuals with endometriosis undergo several condition‐related surgeries, and yet still require analgesics or hormonal medications 1 year later (Vercellini et al., 2009), with 60% of those taking hormonal medications continuing to experience significant pain (Vercellini et al., 2010). Further mental and physical outcomes worsen as surgeries increase (Capezzuoli et al., 2021). While hormonal medications are enshrined within ESHRE (Becker et al., 2022), American College of Obstetricians and Gynecologists (2010) and NICE (2024) endometriosis guidelines, more accessible and less invasive management options are clearly needed. Psychological interventions are one such option; however, the available evidence regarding their efficacy for endometriosis‐related pain is sparse. Systematic reviews of psychological interventions for endometriosis‐related pain have yielded a limited number of studies, with 10 identified by Samami et al. (2023) and 11 by Van Niekerk et al. (2019). Both reviews called for more high‐quality studies to provide stronger evidence. To support this call, we posit that endometriosis pain experiences must first be more comprehensively understood since this foundational understanding is the platform from which high‐quality, specific, and efficacious psychological interventions can be developed.

Qualitative research is well placed to guide our understanding through gathering information about experience from those who live with it. Some qualitative research on endometriosis‐related pain to date has studied the uncertainty of pain (Denny, 2009), the experience of living with the pain (Denny, 2004), and the complexity of pain (Drabble et al., 2021). The rich descriptions that people with endometriosis use to describe their pain (Bullo, 2020; Bullo & Hearn, 2021) and how these descriptions differ between patients and physicians (Fauconnier et al., 2013) have also been qualitatively investigated and provide meaningful insight into the communication of pain. Beyond a pain‐specific lens, a systematic review of qualitative research investigating the effects of endometriosis on the lives of those living with the condition found pain to be a central concern with a widespread impact on people's quality of life (Young et al., 2015).

This existing research is valuable in enhancing our understanding of this under‐researched yet common experience. However, endometriosis‐related pain is particularly complex due to the heterogeneous relationship between endometriosis lesions and the level of pain and associated impact experienced (Vercellini et al., 2007). This means that individuals can experience similar levels of condition severity, yet different levels of pain, distress, and impact related to their pain. It is likely that psychosocial and biological factors interact to produce such heterogeneous pain experiences, as proposed by the widely accepted biopsychosocial model of pain (Crombez et al., 2012; Engel, 1977; Vlaeyen & Linton, 2000). Indeed, a recent review found that depression, anxiety, and pain catastrophizing are associated with worse endometriosis‐related pain (Kalfas et al., 2022). Additionally, research demonstrates elevated rates of fear of progression among people with endometriosis, which are associated with pain interference (Todd et al., 2023). As such, there is a growing interest in the psychosocial factors involved in endometriosis‐related pain experiences. Despite this, there remains little understanding of the factors that exacerbate or alleviate endometriosis‐related pain impact and distress and how these may interact. This is a crucial gap in the literature given these factors are potential treatment targets for improving pain experiences and psychosocial outcomes.

The aim of the present study was to improve the current understanding of endometriosis pain, particularly the factors associated with elevated pain distress and impact. Using a mixed‐methods approach, we conducted qualitative interviews with people with varying endometriosis pain experiences, which were analysed following grounded theory principles. From this analysis, the COMPASS model was developed. We then content analysed qualitative text responses from a large sample of people living with endometriosis pain regarding their needs and priorities, which we used to cross‐validate and further refine our model.

## METHOD

### Participants

Ethical approval was obtained from the University of Sydney Human Research Ethics Committee (#2021/711). Participants were recruited by advertising through Endometriosis Australia. To be eligible for the study, participants were required to be aged 18 or older, fluent in English, located in Australia, and with laparoscopically diagnosed endometriosis.

### Study design, procedure, and data collection

The study used a mixed‐methods approach to capture a broad range of endometriosis pain experiences. First, participants responded to a study advertisement and followed a link to an online survey, hosted by Qualtrics. Participants provided informed consent before answering quantitative survey questions about their age, gender, endometriosis diagnosis, diagnosis delay, years of pain experienced, days of pain per month, and comorbid conditions. Pain severity and pain‐related distress were assessed using visual analogue scales (0; No pain/No distress, 10; Worst pain/distress imaginable), informed by the Brief Pain Inventory (Cleeland & Ryan, 1994). Pain‐related interference was assessed with the Pelvic Pain Impact Questionnaire (PPIQ) (Chalmers et al., 2017). This quantitative information was not the primary focus of this study; it was obtained to facilitate strategic sampling of participants with different pain severity and interference ratings. A measure of interpretation bias was also included but was not central to the aims of this paper and is reported elsewhere (Pickup et al., 2023). Optional open‐ended survey questions developed for the study then followed, which asked about the needs and priorities of people with endometriosis pain (see Tables S1–S3), to complement qualitative interviews. Survey data were collected in October 2021.

A subset of participants who completed the survey were strategically sampled for interview based on ensuring representation from a broad range of pain experiences, including pain severity, distress and interference ratings and days of pain per month. That is, participants with high pain severity, distress and interference ratings, as well as those with high severity but low to moderate distress and impact were sampled, across a range of days of pain per month (see Table 2). All interviews took place over Zoom between November 2021 and August 2022 and were conducted by the first author. All authors were psychologists and researchers, one of whom with a provisional diagnosis of endometriosis. The interview was piloted with an individual with lived experience, known to the first author. Interviews were guided by a semi‐structured interview schedule and commenced with the open‐ended question “Can you please tell me about your experience of endometriosis‐related pain?”. All interviews were recorded and transcribed verbatim. The transcriptions were then checked against the recordings and analysed. Sampling of participants continued until data saturation was reached (no new themes across three interviews) (Guest et al., 2006) reflecting the iterative nature of grounded theory (Corbin & Strauss, 2008). The interviews ranged in length from 33 to 135 min (*M* = 56). Memos documenting initial ideas were written immediately after each interview.

### Data analysis

Analysis of survey data consisted of sorting pain severity, distress, and interference ratings to identify participants who reported various experiences. Interview data collection and analysis occurred concurrently, consistent with the constant comparative nature of grounded theory (Corbin & Strauss, 2008). Memos were consulted when coding, which facilitated comparison between reflections and interviews, enriching data analysis and guiding further data collection. Transcripts were independently open coded by two authors, with a third author checking a portion. First, initial codes and preliminary themes emerging from the data were identified and compared across interviews. The coders then met to amalgamate ideas, determine lower‐level and higher‐level codes, and combine lower‐level codes into higher‐level codes to form an overarching understanding of the themes in the interviews. The coders then returned to the transcripts to check that this understanding was reflected in the data. The model was then developed to visually represent our grounded theory. This was also cross‐checked against the data. Our analysis resulted in a grounded theory that describes the biopsychosocial processes involved in endometriosis pain, rather than a reiteration of participant experiences. This analysis was enhanced by the authors' clinical psychology experience, which facilitated rich contextualization of interpretations and a formulaic understanding of the themes and processes that arose from the data.

Following the grounded theory analysis and model development, open‐ended survey responses were analysed using content analysis (Krippendorff, 2013). We used the coding framework generated through the analysis of the qualitative interviews and quantified the number of participants whose responses could be categorized by each code. In addition, responses that indicated a theme that had not been identified were coded by the author team, since it was of interest whether in a larger sample, additional themes would emerge. The content analysis findings were used to validate and complement the model.

## RESULTS

### Participant characteristics

A total of 873 participants with endometriosis‐related pain completed the online survey. The demographics and pain outcomes of this sample are presented in Table 1. Most participants (83.2%) reported Australian, Caucasian, or White ethnicity and identified using she/her pronouns (98.2%). A large proportion of participants (41.5%) were unsure of their specific endometriosis diagnosis, while deeply infiltrating endometriosis was the most common diagnosis (31.6%), followed by superficial peritoneal endometriosis (11.1%).

**TABLE 1:** Demographics and pain outcomes of participants who responded to the survey.

Variable	Mean	SD	Range
Age	31.68	7.56	18–62
Pain severity (/10)	7.39	2.34	0–10
Pain impact (/28)	17.15	6.12	0–28
Distress (/10)	5.90	2.25	0–10
Pain days per month	14.61	10.03	0–31
Diagnosis delay (years)	9.47	6.60	0–37
Years in pain	13.21	7.71	1–41

*Note*: *N* = 845–873; Pain impact = PPIQ scores (note: scores/28 due to missing item).

Sixteen participants were strategically sampled from the larger sample for interview. Demographics and pain outcomes of interviewed participants are displayed in Table 2. These participants ranged in age from 20 to 54, and all identified using she/her pronouns, one participant additionally identifying with they/them pronouns. Most participants (94%) reported Australian, Caucasian, or Anglo‐Saxon ethnicity. Half of these participants were unsure of their specific endometriosis diagnosis, while of those with a specific diagnosis, deeply infiltrating endometriosis was the most common.

**TABLE 2:** Demographics and pain outcomes of interview sample.

ID	Age	Pronoun	Ethnicity	Diagnosis	Diagnosis delay (years)	Worst pain	Pain days	Distress	PPIQ
1	25	She/her	Australian	Unsure	8	10	30	4.8	7
2	31	She/her; they/them	Caucasian	Superficial peritoneal endometriosis	14	10	30	9	28
3	54	She/her	Caucasian	Deeply infiltrating endometriosis	17	0.9	2	8.5	14
4	35	She/her	Anglo	Unsure	15	2.6	1	2.9	6
5	36	She/her	Australian	Unsure	17	5	28	5	18
6	42	She/her	Australian	Deeply infiltrating endometriosis	8	10	2	8.2	28
7	41	She/her	Australian	Deeply infiltrating endometriosis	19	10	31	3	10
8	31	She/her	Australian	Superficial peritoneal endometriosis	10	8.2	30	10	28
9	36	She/her	Australian	Unsure	18	8	4	2	9
10	20	She/her	Australian	Unsure	6	8.5	4	3	23
11	25	She/her	Australian	Unsure	7	8.6	26	3.1	24
12	30	She/her	Caucasian	Deeply infiltrating endometriosis	12	10	4	10	8
13	28	She/her	Australian	Deeply infiltrating endometriosis	11	7.2	18	4	8
14	27	She/her	Australian	Unsure	9	6	20	6	15
15	39	She/her	Caucasian	Other	8	10	14	3.8	14
16	30	She/her	Mixed	Unsure	11	9.5	31	2.5	20

*Note*: ID = participant ID, pain days = number of endometriosis‐related pain days in a month, Distress = pain‐related distress rating.

Abbreviation: PPIQ, pelvic pain impact questionnaire score (/28 due to missing item).

A total of 841 participants from the larger sample also completed the optional open‐ended survey responses and were included in the content analysis. Those who left open‐ended responses did not differ from those who did not in terms of pain distress, severity, and interference; see Table S4.

### Grounded theory findings

Six major themes related to pain experiences emerged from the grounded theory‐informed analysis of the 16 interviews. These are described below with participant quotes to substantiate the analysis.

#### Gendered nature of endometriosis and pain

The effects of endometriosis and pelvic pain as gendered experiences were apparent. Participants indicated that their pain, particularly pelvic pain, was commonly underestimated or misconstrued as normal based on their gender. These experiences were particularly salient in healthcare settings, where participants felt male and female doctors often did not believe their pain reports:

I was like here's something else going on here, or like there's a bigger problem and my doctor was like no like you're a girl just get over it, everyone goes through it. (#1)

You go to the doctor when you're younger and they just say oh, you know pain is normal with periods…. (#11)

#### Invalidation

As a result of the gendered nature of endometriosis pain, participants described pervasive experiences of invalidation from healthcare professionals, family, and friends. These experiences and subsequent effects are described below.

##### Diagnosis delay

All interviewed participants described a several‐year delay between endometriosis symptom onset and diagnosis, consistent with the 9.47‐year average diagnosis delay in the wider sample. Participants described that their pain and symptoms were dismissed and normalized by healthcare professionals and loved ones during delays, consistent with the gendered nature of their pain described above. Participants described internalizing invalidating sentiments, which discouraged them from seeking a diagnosis. Some participants described grief around what life could have been like if diagnosed earlier:

Had I just been diagnosed earlier like would I just been you know able have kids naturally not being through so many surgeries… I always think of how life could have been a bit different… it's hard. (#12)

##### Lack of knowledge and understanding about endometriosis

Participants felt that the gendered nature of endometriosis pain contributed to the invalidation they experienced, but the fact that endometriosis is generally poorly understood in terms of knowledge available about the condition, as well as healthcare professionals and the wider community not being up to date with the available knowledge, also played a role in their negative experiences. Notably, most participants felt that healthcare professionals lacked an understanding of the available knowledge. For example, participants described experiences of doctors being unable to answer questions and instead instructing them to do their own research:

The doctor that did my first laparoscopy was just like oh you've got this (endometriosis) you know read about it. It should be fine now we've cut it out, and I said oh, what about fertility, what about this and that, she's just like oh I don't know do a bit of research. (#13)

Even when healthcare professionals did provide the available information, this information was still limited due to the prevailing lack of knowledge about the condition:

…he (an excision specialist) gives me like much more information … but still it's a bit disheartening… they don't know what causes it they don't know really how to fix it. (#13)

Most participants described that the lack of understanding from family, friends or romantic partners had impacted their relationships and often left them feeling isolated. One participant described the lack of understanding as the most difficult aspect of endometriosis:

The general conception… that it's just a bad period… I think that is the biggest struggle that I faced, and in terms of how it affects my pain, it almost feels like I can't show the full extent of my pain, because people might not really grasp what it means. (#14)

##### Healthcare experiences

The invalidation that participants described in their home life was also commonly experienced in the healthcare system. All but one participant described feeling dismissed by healthcare professionals from their teenage years when first seeking help for symptoms. Some participants felt invalidated when diagnosed. This invalidation seemed to stem from the manner in which the diagnosis was provided, a poor or limited explanation about the condition, and often the absence of an ongoing management plan:

When I woke up, the first thing she (the gynaecologist) said to me was, well, I don't know why you're in so much pain, because it wasn't that bad… The aftercare was like pretty much non‐existent like I went and had a follow up with her, but she was just like, yeah, you have endo, and that was kind of it. (#14)

Some participants described feeling dismissed when attending emergency departments in severe pain. These experiences resulted in participants waiting until symptoms were extremely severe before seeking help or avoiding seeking medical assistance in the future:

I went into an emergency department and I had to lie on the table for like two hours in agony, and the doctor just kept coming in and out and saying, there's nothing physically wrong with you… now, if I get really bad my fiancé is like, okay, let's go to hospital or let's go to the doctor, and I'm still like no, because I've still got this fear of not being believed and having them treat me like a drug addict. (#15)

Invalidating experiences within the healthcare system were described by some participants to exacerbate stress and, consequently, pain levels:

I know that going to the doctors and coming out crying because you feel like they don't care or don't listen to what you have to say makes me emotional and therefore gives me more pain. (#11)

In contrast to invalidating experiences, some participants described the positive effects of feeling validated in healthcare settings. These experiences seemed to be born from interactions with healthcare professionals who were educated about endometriosis, who ensured that their patients understood, listened to patient experiences, and answered their questions:

The doctors … were able to explain things to me in a way that allowed you to put the pieces together, and they also created that safe space to ask questions… you feel empowered by that. (#5)

#### Distrust in healthcare system

Although some people had found empathic healthcare professionals, all but one participant conveyed some distrust of the healthcare system. This arose from invalidating experiences, long diagnosis delays and feeling that healthcare professionals lacked understanding about endometriosis. Distrust could result in participants “quizzing” doctors about endometriosis and discontinuing the interaction if they perceived their knowledge was inadequate:

If I go to a senior doctor… I put them through a short quiz on how well they actually know their stuff and if they are floundering then I don't give much time. (#3)

Despite pervasive experiences of invalidation, most participants had found healthcare professionals whom they trusted. While being relieved, participants emphasized how the searching process was nevertheless burdensome:

It was a long time before I found people that actually listened, and I think I came away from a lot of those appointments feeling really violated or dismissed. (#16)

#### Burden

All interviewed participants described the heavy burden associated with endometriosis, consistent with the average pain impact and frequency scores in the wider sample. This arose from the wide range of endometriosis symptoms, comorbid mental health difficulties, and pain, which impacted several domains of functioning. These symptoms included fatigue, heavy bleeding, nausea, bloating, sleep disturbance and brain fog:

It's more than just period pain, like it sort of affects every aspect of your life in terms of pain, but also it obviously affects your life more than just pain… things like headaches, migraines that are linked to a flare up … nausea, fatigue, not being able to do things, sleep disturbance from being in discomfort… and recognising the emotional impact of going through that as well. (#10)

Some participants also described the negative financial impacts of living with a chronic condition, including the cost of appointments, medications, and time off work. Most participants also described the emotional toll of fertility difficulties and role changes in work, family, and social life. Most participants had either forgone their fertility in the pursuit of pain elimination or had considered it, demonstrating the significant burden and impact of pain.

#### Sense making

Participants seemed to engage in various strategies to make sense of their condition, particularly after living in uncertainty and feeling unable to rely on healthcare professionals for answers for several years. Some strategies also seemed to reduce uncertainty, move towards acceptance, and aid communication about endometriosis and its impacts.

##### Imagery and physicality of language

Participants described their pain sensations with rich biomedical imagery and analogies that conjured imagery. These descriptions seemed to function to communicate the severity of one's pain, after long histories of invalidation. Participants were not always visibly distressed when describing pain using analogies; however, the vivid and physical nature of these descriptions implicitly conveyed distress:

It feels like there's like a hook inside my vagina that's just being like yanked down. (#14)

Biomedical imagery involved participants interpreting their pain as being the result of anatomical/structural abnormalities, often integrating doctors' communications regarding the location and characteristics of the endometriosis:

I have pretty consistent lower back pain on my right‐hand side, my surgeon thinks that that's because of my ovary being stuck. (#14)

##### Pain interpretations

Participants interpreted pain in various ways. Participants who used biomedical imagery tended to interpret their pain as having a biomedical cause:

I have in pain my left ovary, that's where the majority of my pain is… I know that one is completely stuck to everything around it… (#2)

This interpretation style could reduce uncertainty about pain that had accumulated during long diagnosis delays, however for others, interpreting pain as having a biomedical cause, for which management options are limited, exacerbated pain outcomes.

Some participants, often those who had been provided education about the biopsychosocial model of pain, interpreted their pain experiences as a combination of biological, psychological, and social (biopsychosocial) factors. These participants were aware of the bidirectional link between their psychological states and pain experiences:

Yeah, if I'm very stressed, or if I'm very tired I'm definitely more likely to experience pain… when I'm feeling anxious, I'm definitely hyper aware of it (the pain). (#10)

Participants with a biopsychosocial understanding of their pain often make less catastrophic interpretations of their pain:

To know what it is and why it's happening allows you to kind of go, this is okay, you know nothing, nothing bad is going to happen… (#5)

Further, less catastrophic interpretations of pain facilitated pain management:

Perhaps when I was younger (in a flare) I'd be like oh, this is terrible, I'm going to be in bed, I hate this, I'm stuck like this forever, it's the worst thing ever. Now I just go oh well I've done this 100 times before, I can do it. (#13)

##### Identity

Participants had various ways of identifying with their condition and life more broadly. Some participants identified with endometriosis and organized their lives around trying to prevent or cope with pain:

It literally feels like my whole life is just made trying to prevent this pain that I'm potentially going to be in. (#11)

Participants identifying in this way tended to focus more on their symptoms and had less engagement with activities of enjoyment. Together, these effects culminated in greater pain‐related impact and distress and a less felt sense of agency. other participants fostered an identity that aligned with personal values, goals, and interests, beyond endometriosis. This was protective for coping through facilitating engagement in activities of enjoyment and, in turn, alleviating pain‐related distress and impact:

I love creating things. So, if I make time to write, or to paint, or something, things that are kind of life‐giving, make pain more manageable… Sometimes it feels like the pain steals my life or steals me. Whereas those things make me feel more like myself. (#16)

Exercise was a common activity of personal value that participants described as protective for mental and physical health and pain management:

It's been really figuring out and having consistent exercise that is gentle on my body helps (to manage pain and distress). (#16)

Many participants described having a “high pain threshold” or that they were “strong”, indicating a felt sense of resilience. Some participants attributed this to dealing with pain without answers or treatment during long diagnosis delays. Most participants with a felt sense of resilience tended to experience less pain‐related impact and distress.

I had my baby recently and I didn't have an epidural like this is what I deal with, this pain… I do have a very high pain threshold… (#12)

#### Agency

Participants' perceptions of or ability to exercise agency seemed to buffer against pain‐related distress and impact. Agency is referred to here as one's ability to exercise choice and control over their actions. The various aspects of agency that emerged from the interviews are outlined below.

##### Health literacy

Participants' health literacy, that is their ability to access, understand, and think critically about endometriosis‐related information, influenced their agency. Participants described pursuing various avenues of endometriosis‐related information. Some participants described consulting research and evidence‐based guidelines, while others described how social media groups were helpful. Some participants, however, indicated that online spaces could be unhelpful in reinforcing a pain‐centric identity. Health literacy could also be positively influenced by treatment and resources from healthcare professionals. Regardless of information source, greater health literacy seemed to reduce uncertainty, aid informed decision making, and facilitate agency. For example, one participant spoke of increased understanding facilitating pain management:

There was a book that they (physiotherapist) gave me that was really helpful, I think just understanding … how pain works…there are a whole heap of different things going on that are contributing to the pain. (#16)

##### Supportive multidisciplinary team

The involvement of supportive health professionals, particularly a multidisciplinary team (MDT), facilitated endometriosis care, especially through pain management plans. These plans could include pain relief and hormonal medications, physiotherapy exercises and stretches, dietetics input, and psychological strategies. A supportive MDT and plan helped participants to feel validated and reduced the pain impact and their uncertainty and fear regarding pain management, which in turn bolstered agency.

My symptoms are much the same as they were when I first got it when I was 12 but they just don't get in my way as much anymore, because I have like strategies for coping with it… I have all this wonderful support network… I went to a pain specialist, and we did all kinds of things like food not to eat when I'm flaring, different supplements, different medications and CBT was like a part of that pain plan that we came to together… physio has (also) helped a bit… (#13)

##### Workplace support and flexibility

A few participants described that when they had communicated directly with their workplace regarding their symptoms and needs, this resulted in increased workplace understanding and flexibility (i.e., working from home allowances or flexible hours), their sense of autonomy and agency was enhanced, and the pain impact was reduced:

The flexibility of work now, I can break up my day if I want … the ability to just be like I'm not going into the office today, the pain has less of an impact. (#5)

##### Acceptance

Participants who were less accepting of endometriosis tended to have a greater focus on their pain and efforts to eliminate it, to the detriment of engaging in other valued activities, consistent with a pain‐centric identity. This tended to be associated with a less felt sense of agency, due to feeling like pain controlled their lives. In contrast, participants who were more accepting of endometriosis seemed more able to focus on other valued and rewarding aspects of life, reporting greater agency, which was consistent with a life‐centric identity. Participants acknowledged that acceptance mostly came with time.

I've definitely been there in the “why me”, it's like grieving the loss of who you are… but definitely the acceptance… putting it out there and saying, this is who I am… pushing myself to do other things in life and realising that I can live with endometriosis and do other things in the world. So, it's not just one thing that's defining me. (#15)

##### Development of the COMPASS model

**FIGURE 1:**
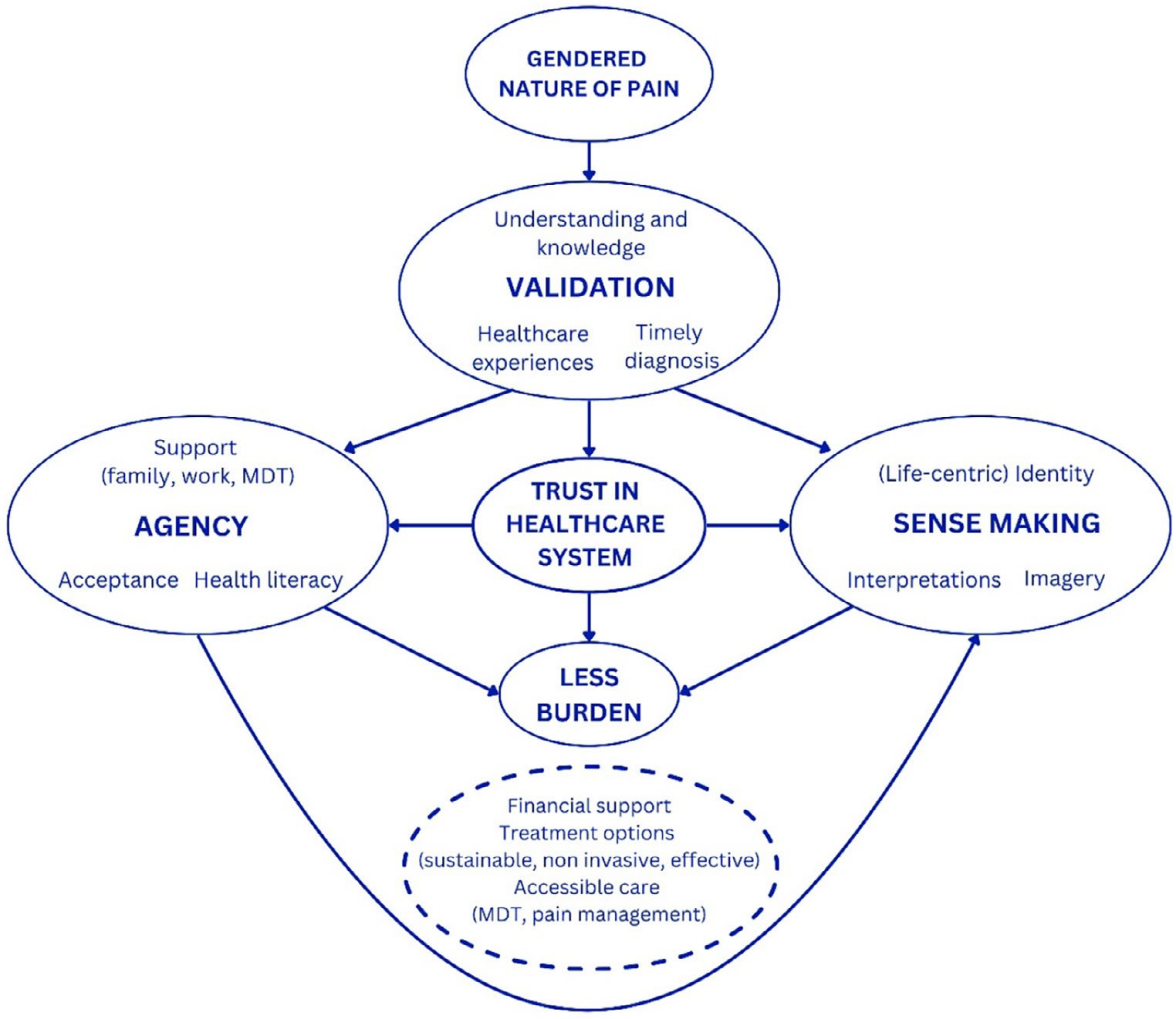
The COMPASS model representing the relationships between themes of endometriosis‐related pain experiences associated with less pain‐related distress and impact. Additional systemic factors identified through content analysis are depicted by the dashed line box.

From our grounded theory informed analysis of interview data, we developed a novel hypothesis‐generating model of endometriosis pain. Since this research ultimately aims to contribute to enhanced care in endometriosis, our model intentionally demonstrates the pathways to better outcomes for people with endometriosis (see Figure 1). Despite the gendered nature of pain and invalidation being almost universally experienced, our model proposes that validation can be experienced, particularly through healthcare professionals who believe and listen to their patients. Validation is proposed to foster trust and is situated as the platform from which agency and sense‐making can develop. Agency and sense‐making can be mutually reinforcing such that participating in value‐based rewarding activities and building an identity beyond endometriosis pain can facilitate acceptance, which further facilitates identity building. Together, these processes can culminate in reduced pain distress, impact, and burden, even in the face of high pain severity.

Alternatively, the model can also depict the processes associated with greater pain impact and distress, as displayed in Figure S1.

##### Content analysis findings

Open‐ended survey responses from 841 participants were content analysed. This analysis was used as additional verification of the grounded theory themes and model. Common codes, with frequency counts, are reported below, with additional findings in Tables S1–S3.

##### Invalidation

Consistent with interviews, invalidation was the most frequent code identified in responses regarding the greatest challenge faced by people with endometriosis, indicated by 428 participants. Responses such as “being dismissed when in pain, being gaslit by medical professionals” and “being dismissed” were coded as invalidation, in line with symptom invalidation literature (Bontempo, 2022; Kool et al., 2010; Nicola et al., 2021).

Invalidation from healthcare professionals specifically was noted by 132 participants. The lack of understanding from others was the second most common code identified regarding the greatest challenge of endometriosis, indicated by 316 participants. Consistent with our model, 542 participants suggested more education of and understanding from others about endometriosis could make a difference. Notably, more education for healthcare professionals was suggested to improve pain management. Obtaining an endometriosis diagnosis was the third most common code regarding the greatest challenge of endometriosis, indicated by 147 participants. Invalidation and lack of understanding about endometriosis were commonly cited reasons for diagnosis delays, consistent with the model.

##### Agency

Content analysis of participants' responses revealed their desire for more support. MDT care was the second most common code identified in responses to the question of how to improve pain management, indicated by 97 participants. This indicates participants' desire for enhanced agency, through feeling better supported to manage pain. The desire for agency through health literacy was apparent as more pain education and self‐management resources were the fifth most common code regarding how to improve pain management, indicated by 54 participants. These findings align with the model.

##### Systemic factors

Other prominent concepts identified through the content analysis relate to systemic factors.

The lack and limitations of treatment options was one systemic factor identified and was the third most common code regarding the greatest challenge of endometriosis, indicated by 152 participants. Better treatment options were the third most common code regarding how to improve pain management and the fifth most common code as to how to improve participants' lives, indicated by 92 and 131 participants, respectively. Access to care was another systemic factor; difficulty accessing treatments and care was the fourth most common code regarding the greatest challenge of endometriosis, indicated by 102 participants. Commonly cited barriers to accessibility included the cost of care, limited specialist availability, and long wait times. Improving care and treatment accessibility was the most common code regarding how to improve pain management and the third most common code regarding how to make a difference for those with endometriosis, indicated by 256 and 162 participants, respectively. Financial support was another factor identified; more financial support was the fourth most common response regarding how to make a difference and improve pain management. Suggested financial support measures included more rebated healthcare appointments and government support payments.

These factors were added to the model to situate pain experiences within broader systemic issues. While almost universally experienced, the effects of these additional factors are likely to vary between individuals. For example, the inaccessibility of care and lack of financial support may have greater impacts on individuals from lower socio‐economic backgrounds or rural areas compared to those with greater financial security or those living close to an MDT pain or endometriosis centre. The lack of non‐invasive treatments without side effects also contributes to the impact of the condition, though side effects may be experienced differently across individuals.

## DISCUSSION

The current study aimed to investigate the psychosocial factors that contribute to worse endometriosis‐related pain distress and impact. We used a mixed‐methods design to develop a grounded theory of endometriosis‐related pain experiences from individual qualitative interviews. From this grounded theory, the novel COMPASS model of endometriosis‐related pain was born, which was additionally validated by content analysis of brief text responses from a large (*N* = 841) sample of people with endometriosis‐related pain.

The experiences and impacts of invalidation were particularly striking. In interviews, participants described feeling dismissed, not believed, not understood, and delegitimised, experiences which align with invalidation as defined in the symptom invalidation literature (Bontempo, 2022; Kool et al., 2010; Nicola et al., 2021). Notably, over 400 participants saw invalidation as the greatest challenge of endometriosis, surpassing pain and the absence of a cure. Experiences of symptom invalidation in endometriosis are widely documented (Facchin et al., 2018; Grundström et al., 2018, 2023; Young et al., 2015, 2020) and often relate to pain. Our findings demonstrate that pain invalidation can exacerbate endometriosis‐related pain outcomes, in line with recent findings that illness invalidation is associated with worse distress (Woldhuis & Gandy, 2024). The current findings and existing literature suggest that people with endometriosis distrust and disengage from the healthcare system if perceiving healthcare professionals to not believe their accounts (McGowan et al., 2007) or lack knowledge about endometriosis (Seear, 2009). That is, if they feel invalidated. In contrast, positive healthcare experiences such as feeling validated can increase self‐management (O'Hara et al., 2019) and reduce distress (Facchin et al., 2018). Of course, the themes regarding the knowledge of healthcare professionals were the perceptions of participants, who expressed that the lack of general knowledge was compounded by healthcare professionals not being up to date with the available knowledge. Interviews with healthcare professionals could help to triangulate perspectives on this issue. Regardless, these findings highlight the importance of validating the experiences of people with endometriosis.

There are various avenues through which validation can be facilitated. One avenue is more research, as with more research comes more knowledge and understanding. The lack of prevailing knowledge about endometriosis is a result of the condition being disproportionately under‐researched, which some suggest is a result of structural, cultural, and political processes, encompassing the gendered nature of the condition and pain (Hudson, 2022; Seear, 2016). Recent action from the Australian Government, including the National Action Plan for Endometriosis (2018) and the announcement of multidisciplinary endometriosis and pelvic pain clinics (2023) are important steps for facilitating research and increasing understanding and validation. Further initiatives like these seem indicated. Another potential avenue for improving validation is through widespread consideration of how early discourse about menstruation born from androcentric medical contexts (Hudson, 2022; Jones, 2015) continues to contribute to stigma about endometriosis (Culley et al., 2013; Sims et al., 2021), biased underestimations of women's pain in healthcare (Schäfer et al., 2016) and community settings (Zhang et al., 2021), and hysteria discourse among medical professionals when treating and discussing patients with endometriosis (Young et al., 2019). Addressing these avenues should be prioritized in the field, particularly since the COMPASS model positions validation as the platform from which agency and sense‐making are developed.

Agency and sense making are core components for alleviating pain‐related distress and impact. Sense making, particularly having an identity centred around personal goals and values, was helpful for alleviating pain‐related distress and impact, despite high pain severity. Recent variations of the fear avoidance model of chronic pain incorporate the person's motivational state and goals (Crombez et al., 2012). Karsdorp and Vlaeyen (2011) found the strong pursuit of achievement or hedonic (pain‐relief) goals was associated with worse pain severity and interference, whereas a more flexible goal pursuit style, including switching between achievement and hedonic goals, related to lower pain severity and interference in people with musculoskeletal pain. We see these findings mirrored in the current study such that participants who prioritized activities of value and enjoyment, in the context of pain management, tended to experience less pain‐related impact and distress. Orienting goals away from pain elimination to other rewarding aspects of life is a core tenet of pain acceptance (McCracken et al., 2004), where pain acceptance refers to living alongside pain without trying to reduce or respond to it (McCracken, 1998). Acceptance of chronic pain has been found to uniquely contribute to improved well‐being (Viane et al., 2003), while acceptance of endometriosis has been associated with improved quality of life (Bień et al., 2020). Therefore, facilitating acceptance, potentially through pursuing goals and an identity of personal value, could improve well‐being for people with endometriosis.

### Implications

To our knowledge, we present the first model of endometriosis‐related pain. The leading model of pain, the fear avoidance model (Crombez et al., 2012; Meulders, 2019), suggests that people interpret pain as signifying harm, which drives a vicious cycle of attention to pain, attempts to control pain, avoidance of activity, negative affect, pain interference, and worsening pain. Alappattu and Bishop (2011) reviewed the relevance of certain aspects of the fear avoidance model to pelvic pain but acknowledged important caveats to its applicability, including differences in pain origins and sequelae. Our model did find that biomedical interpretations seemed to contribute to pain experiences, while prioritizing valued life goals in the context of pain management was protective for coping, in line with the fear avoidance model (Crombez et al., 2012; Meulders, 2019). However, our model was novel in finding the involvement of several other psychological factors, including invalidation, imagery, identity, health literacy, and distrust in healthcare professionals, which impacted pain coping. Further, our model was novel in including systemic factors such as the gendered nature of pain and the accessibility of care, which can contribute to pain experiences, as recognized in the biopsychosocial model of pain (Engel, 1977).

The COMPASS model provides several potential psychosocial treatment targets and thereby supports recent calls for enhanced interdisciplinary care that treats the biopsychosocial needs of people with endometriosis (Evans et al., 2022; Rowe et al., 2021; Young et al., 2017). First and foremost, the gendered nature of endometriosis and women's pain, along with the current level of understanding about endometriosis must be addressed so that validation can improve. Second, bolstering individuals' sense of agency and assisting with sense‐making are important and interrelated treatment targets for reducing pain distress and impact. That is, helping one to feel more supported and better understand their condition is likely to help with acceptance and building an identity around one's values and interests, beyond endometriosis. Third, educating people with endometriosis about the biopsychosocial nature of pain and providing evidence‐based self‐management and treatment resources could enhance health literacy and pain management and therefore facilitate pain coping (Mardon et al., 2023). Additionally, increasing the accessibility of effective treatment options, particularly MDT management, which is currently the most evidence‐based approach (Ugwumadu et al., 2017), is crucial for improving endometriosis pain management.

### Limitations, strengths and future directions

This study is not without limitations. All participants were recruited through Endometriosis Australia, and therefore may be more help seeking and distressed than those not engaged with endometriosis organizations. Further, most participants were White, which may limit the generalizability of these findings, particularly since individuals from diverse backgrounds tend to experience more complex intersectional stigma (Turan et al., 2019). Future research could investigate whether the biopsychosocial factors of endometriosis pain differ for people from different backgrounds.

This study also had several strengths. To our knowledge, the COMPASS model is the first biopsychosocial model of endometriosis‐related pain and is therefore an important contribution to the field. Second, our model provides several potential psychosocial intervention targets for improving outcomes for individuals with endometriosis. These potential targets are particularly valuable considering the lack of quality evidence‐based psychological interventions for endometriosis (Van Niekerk et al., 2019) and the limited efficacy of current biomedical treatments (Becker et al., 2017; Singh et al., 2020). Third, while member checking was not conducted, invalidation and agency, two major components of the model, were validated by the text responses of a broad sample of 841 participants with endometriosis‐related pain. Future research to empirically validate the model is indicated and could focus on establishing temporal or causal relationships between constructs.

## CONCLUSION

This study presents a new grounded theory‐informed understanding of the complex psychosocial factors that influence endometriosis‐related pain. The relationships between these factors were generated by in‐depth qualitative interviews and are depicted in a hypothesis‐generating model that could be integrated into MDT care, following empirical validation. This model has been further enhanced through the analysis of text responses from a large sample of people with endometriosis, which highlighted the wider system factors that must be considered as part of any research or clinical progress. We consider the outcomes of this study as a call to action, to (a) address invalidation within healthcare systems, (b) prioritize research and implementation of effective endometriosis treatments, and (c) better educate health professionals and the public about endometriosis. With a whole‐systems approach, endometriosis pain can be better managed to improve outcomes for the large proportion of the population who have this condition.

## AUTHOR CONTRIBUTIONS

**Brydee Pickup:** Conceptualization; methodology; investigation; validation; visualization; writing – original draft; software; formal analysis; project administration; data curation. **Louise Sharpe:** Conceptualization; methodology; supervision; validation; writing – review and editing. **Jemma Todd:** Conceptualization; funding acquisition; investigation; writing – review and editing; validation; methodology; formal analysis; supervision; resources.

## Supporting information


Table S1.
Figure S1.

## Data Availability

The data that support the findings of this study are available on request from the corresponding author. The data are not publicly available due to privacy or ethical restrictions.

## References

[bjhp12794-bib-0001] Alappattu, M. J. , & Bishop, M. D. (2011). Psychological factors in chronic pelvic pain in women: Relevance and application of the fear‐avoidance model of pain. Physical Therapy, 91(10), 1542–1550. 10.2522/ptj.20100368 21835893 PMC3185223

[bjhp12794-bib-0002] American College of Obstetricians and Gynecologists . (2010). Practice bulletin No. 114: Management of Endometriosis. Obstetrics & Gynecology, 116(1), 223–236. 10.1097/AOG.0b013e3181e8b073 20567196

[bjhp12794-bib-0003] Australian Government Department of Health and Aged Care . (2018). National action plan for endometriosis.

[bjhp12794-bib-0005] Becker, C. M. , Bokor, A. , Heikinheimo, O. , Horne, A. , Jansen, F. , Kiesel, L. , King, K. , Kvaskoff, M. , Nap, A. , Petersen, K. , Saridogan, E. , Tomassetti, C. , van Hanegem, N. , Vulliemoz, N. , & Vermeulen, N. (2022). ESHRE guideline: endometriosis. Human Reproduction Open, 2022, hoac009. 10.1093/hropen/hoac009 35350465 PMC8951218

[bjhp12794-bib-0006] Becker, C. M. , Gattrell, W. T. , Gude, K. M. , & Singh, S. S. (2017). Reevaluating response and failure of medical treatment of endometriosis: A systematic review. Fertility and Sterility, 108(1), 125–136. 10.1016/j.fertnstert.2017.05.004 28668150 PMC5494290

[bjhp12794-bib-0007] Bień, A. , Rzońca, E. , Zarajczyk, M. , Wilkosz, K. , Wdowiak, A. , & Iwanowicz‐Palus, G. (2020). Quality of life in women with endometriosis: A cross‐sectional survey. Quality of Life Research, 29(10), 2669–2677. 10.1007/s11136-020-02515-4 32356276 PMC7561574

[bjhp12794-bib-0008] Bontempo, A. C. (2022). The need for a standardized conceptual term to describe invalidation of patient symptoms. Journal of Health Psychology, 27(9), 2104–2114. 10.1177/13591053211024718 34111987

[bjhp12794-bib-0009] Bulletti, C. , Coccia, M. E. , Battistoni, S. , & Borini, A. (2010). Endometriosis and infertility. Journal of Assisted Reproduction and Genetics, 27(8), 441–447. 10.1007/s10815-010-9436-1 20574791 PMC2941592

[bjhp12794-bib-0010] Bullo, S. (2020). “I feel like I'm being stabbed by a thousand tiny men”: The challenges of communicating endometriosis pain. Health, 24(5), 476–492. 10.1177/1363459318817943 30782020

[bjhp12794-bib-0011] Bullo, S. , & Hearn, J. H. (2021). Parallel worlds and personified pain: A mixed‐methods analysis of pain metaphor use by women with endometriosis. British Journal of Health Psychology, 26(2), 271–288. 10.1111/bjhp.12472 32920887

[bjhp12794-bib-0012] Capezzuoli, T. , Vannuccini, S. , Mautone, D. , Sorbi, F. , Chen, H. , Reis, F. M. , Ceccaroni, M. , & Petraglia, F. (2021). Long‐term hormonal treatment reduces repetitive surgery for endometriosis recurrence. Reproductive Biomedicine Online, 42(2), 451–456. 10.1016/j.rbmo.2020.09.018 33277193

[bjhp12794-bib-0013] Chalmers, K. J. , Catley, M. J. , Evans, S. F. , & Moseley, G. L. (2017). Clinical assessment of the impact of pelvic pain on women. Pain (Amsterdam), 158(3), 498–504. 10.1097/j.pain.0000000000000789 28135211

[bjhp12794-bib-0014] Cleeland, C. S. , & Ryan, K. M. (1994). Pain assessment: Global use of the brief pain inventory. Annals of the Academy of Medicine, Singapore, 23(2), 129–138. 8080219

[bjhp12794-bib-0015] Corbin, J. M. , & Strauss, A. (2008). Basics of qualitative research: Techniques and procedures for developing grounded theory. SAGE Publications Inc. 10.4135/9781452230153

[bjhp12794-bib-0016] Crombez, G. , Eccleston, C. , Van Damme, S. , Vlaeyen, J. W. , & Karoly, P. (2012). Fear‐avoidance model of chronic pain: The next generation. The Clinical Journal of Pain, 28(6), 475–483. 10.1097/AJP.0b013e3182385392 22673479

[bjhp12794-bib-0017] Culley, L. , Law, C. , Hudson, N. , Denny, E. , Mitchell, H. , Baumgarten, M. , & Raine‐Fenning, N. (2013). The social and psychological impact of endometriosis on women's lives: A critical narrative review. Human Reproduction Update, 19(6), 625–639. 10.1093/humupd/dmt027 23884896

[bjhp12794-bib-0019] Denny, E. (2004). Women's experience of endometriosis. Journal of Advanced Nursing, 46(6), 641–648. 10.1111/j.1365-2648.2004.03055.x 15154905

[bjhp12794-bib-0020] Denny, E. (2009). I never know from one day to another how I will feel: Pain and uncertainty in women with endometriosis. Qualitative Health Research, 19(7), 985–995. 10.1177/1049732309338725 19470614

[bjhp12794-bib-0021] Drabble, S. J. , Long, J. , Alele, B. , & O'Cathain, A. (2021). Constellations of pain: A qualitative study of the complexity of women's endometriosis‐related pain. British Journal of Pain, 15(3), 345–356. 10.1177/2049463720961413 34377460 PMC8339952

[bjhp12794-bib-0022] Dunselman, G. A. , Vermeulen, N. , Becker, C. , Calhaz‐Jorge, C. , D'Hooghe, T. , Bie, B. D. , Heikinheimo, O. , Horne, A. W. , Kiesel, L. , Nap, A. , Prentice, A. , Saridogan, E. , Soriano, D. , & Nelen, W. L. D. M. (2014). ESHRE guideline: Management of women with endometriosis. Human Reproduction, 29(3), 400–412. 10.1093/humrep/det457 24435778

[bjhp12794-bib-0023] Engel, G. L. (1977). The need for a new medical model: A challenge for biomedicine. Science, 196(4286), 129–136. 10.1126/science.847460 847460

[bjhp12794-bib-0024] Evans, S. , Dowding, C. , Olive, L. , Payne, L. A. , Druitt, M. , Seidman, L. C. , Skvarc, D. , & Mikocka‐Walus, A. (2022). Pain catastrophizing, but not mental health or social support, is associated with menstrual pain severity in women with dysmenorrhea: A cross‐sectional survey. Psychology, Health & Medicine, 27(6), 1410–1420. 10.1080/13548506.2021.1948581 34190659

[bjhp12794-bib-0025] Evans, S. F. , Brooks, T. A. , Esterman, A. J. , Hull, M. L. , & Rolan, P. E. (2018). The comorbidities of dysmenorrhea: A clinical survey comparing symptom profile in women with and without endometriosis. Journal of Pain Research, 11, 3181–3194. 10.2147/jpr.S179409 30588070 PMC6300370

[bjhp12794-bib-0026] Facchin, F. , Barbara, G. , Saita, E. , Mosconi, P. , Roberto, A. , Fedele, L. , & Vercellini, P. (2015). Impact of endometriosis on quality of life and mental health: Pelvic pain makes the difference. Journal of Psychosomatic Obstetrics & Gynecology, 36(4), 135–141. 10.3109/0167482X.2015.1074173 26328618

[bjhp12794-bib-0027] Facchin, F. , Saita, E. , Barbara, G. , Dridi, D. , & Vercellini, P. (2018). “Free butterflies will come out of these deep wounds”: A grounded theory of how endometriosis affects women's psychological health. Journal of Health Psychology, 23(4), 538–549. 10.1177/1359105316688952 28810386

[bjhp12794-bib-0028] Fauconnier, A. , Staraci, S. , Huchon, C. , Roman, H. , Panel, P. , & Descamps, P. (2013). Comparison of patient‐ and physician‐based descriptions of symptoms of endometriosis: A qualitative study. Human Reproduction, 28(10), 2686–2694. 10.1093/humrep/det310 23900205

[bjhp12794-bib-0029] Gambadauro, P. , Carli, V. , & Hadlaczky, G. (2019). Depressive symptoms among women with endometriosis: A systematic review and meta‐analysis. American Journal of Obstetrics and Gynecology, 220(3), 230–241. 10.1016/j.ajog.2018.11.123 30419199

[bjhp12794-bib-0030] Giudice, L. C. , & Kao, L. C. (2004). Endometriosis. The Lancet (British Edition), 364(9447), 1789–1799. 10.1016/S0140-6736(04)17403-5 15541453

[bjhp12794-bib-0031] Grundström, H. , Alehagen, S. , Kjølhede, P. , & Berterö, C. (2018). The double‐edged experience of healthcare encounters among women with endometriosis: A qualitative study. Journal of Clinical Nursing, 27(1–2), 205–211. 10.1111/jocn.13872 28493635

[bjhp12794-bib-0032] Grundström, H. , Engman, L. , Rimhagen, E. , Söderstierna, C. , & Flink, I. (2023). Experiences of communication in women with endometriosis: Perceived validation and invalidation in different contexts, and associations with health‐related quality of life. Journal of Psychosomatic Obstetrics and Gynaecology, 44(1), 2264483. 10.1080/0167482X.2023.2264483 37787069

[bjhp12794-bib-0033] Guest, G. , Bunce, A. , & Johnson, L. (2006). How many interviews are enough?: An experiment with data saturation and variability. Field Methods, 18(1), 59–82. 10.1177/1525822x05279903

[bjhp12794-bib-0034] Hudson, N. (2022). The missed disease? Endometriosis as an example of ‘undone science’. Reproductive Biomedicine & Society Online, 14, 20–27. 10.1016/j.rbms.2021.07.003 34693042 PMC8517707

[bjhp12794-bib-0035] Jones, C. E. (2015). Wandering wombs and “female troubles”: The hysterical origins, symptoms, and treatments of endometriosis. Women's Studies, 44(8), 1083–1113. 10.1080/00497878.2015.1078212

[bjhp12794-bib-0036] Kalfas, M. , Chisari, C. , & Windgassen, S. (2022). Psychosocial factors associated with pain and health‐related quality of life in endometriosis: A systematic review. European Journal of Pain, 26(9), 1827–1848. 10.1002/ejp.2006 35802060 PMC9543695

[bjhp12794-bib-0037] Karsdorp, P. A. , & Vlaeyen, J. W. S. (2011). Goals matter: Both achievement and pain‐avoidance goals are associated with pain severity and disability in patients with low back and upper extremity pain. Pain (Amsterdam), 152(6), 1382–1390. 10.1016/j.pain.2011.02.018 21392886

[bjhp12794-bib-0038] Kool, M. B. , Middendorp, H. V. , Lumley, M. A. , Schenk, Y. , Jacobs, J. W. G. , Bijlsma, J. W. J. , & Geenen, R. (2010). Lack of understanding in fibromyalgia and rheumatoid arthritis: The illness invalidation inventory (3*I). Annals of the Rheumatic Diseases, 69(11), 1990–1995. 10.1136/ard.2009.123224 20498203

[bjhp12794-bib-0039] Krippendorff, K. (2013). Content analysis: An introduction to its methodology (3rd ed.). Sage.

[bjhp12794-bib-0072] Mardon, A. K. , Leake, H. B. , Hayles, C. , Henry, M. L. , Neumann, P. B. , Moseley, G. L. , & Chalmers, K. J. (2023). The efficacy of self‐management strategies for females with endometriosis: A systematic review. Reproductive Sciences, 30(2), 390–407. 35488093 10.1007/s43032-022-00952-9 PMC9988721

[bjhp12794-bib-0040] McCracken, L. M. (1998). Learning to live with the pain: Acceptance of pain predicts adjustment in persons with chronic pain. Pain, 74(1), 21–27. 10.1016/S0304-3959(97)00146-2 9514556

[bjhp12794-bib-0041] McCracken, L. M. , Carson, J. W. , Eccleston, C. , & Keefe, F. J. (2004). Acceptance and change in the context of chronic pain. Pain, 109(1), 4–7. 10.1016/j.pain.2004.02.006 15082120

[bjhp12794-bib-0042] McGowan, L. , Luker, K. , Creed, F. , & Chew‐Graham, C. A. (2007). ‘How do you explain a pain that can't be seen?’: The narratives of women with chronic pelvic pain and their disengagement with the diagnostic cycle. British Journal of Health Psychology, 12(2), 261–274. 10.1348/135910706X104076 17456285

[bjhp12794-bib-0043] Meulders, A. (2019). From fear of movement‐related pain and avoidance to chronic pain disability: A state‐of‐the‐art review. Current Opinion in Behavioral Sciences, 26, 130–136. 10.1016/j.cobeha.2018.12.007

[bjhp12794-bib-0045] National Institue for Health and Care Excellence . (2024). Endometriosis: Diagnosis and management: NICE guideline [NG73]. https://www.nice.org.uk/guidance/ng73 38815122

[bjhp12794-bib-0046] Nicola, M. , Correia, H. , Ditchburn, G. , & Drummond, P. (2021). Invalidation of chronic pain: A thematic analysis of pain narratives. Disability and Rehabilitation, 43(6), 861–869. 10.1080/09638288.2019.1636888 31290347

[bjhp12794-bib-0047] O'Hara, R. , Rowe, H. , & Fisher, J. (2019). Self‐management in condition‐specific health: A systematic review of the evidence among women diagnosed with endometriosis. BMC Womens Health, 19(1), 80. 10.1186/s12905-019-0774-6 31216998 PMC6585070

[bjhp12794-bib-0048] Pickup, B. , Sharpe, L. , & Todd, J. (2023). Interpretation bias in endometriosis‐related pain. Pain (Amsterdam), 164(10), 2352–2357. 10.1097/j.pain.0000000000002946 37326698

[bjhp12794-bib-0049] Rowe, H. J. , Hammarberg, K. , Dwyer, S. , Camilleri, R. , & Fisher, J. R. W. (2021). Improving clinical care for women with endometriosis: Qualitative analysis of women's and health professionals' views. Journal of Psychosomatic Obstetrics and Gynaecology, 42(3), 174–180. 10.1080/0167482X.2019.1678022 31691598

[bjhp12794-bib-0050] Rowlands, I. J. , Abbott, J. A. , Montgomery, G. W. , Hockey, R. , Rogers, P. , & Mishra, G. D. (2021). Prevalence and incidence of endometriosis in Australian women: A data linkage cohort study. BJOG: An International Journal of Obstetrics and Gynaecology, 128(4), 657–665. 10.1111/1471-0528.16447 32757329

[bjhp12794-bib-0051] Samami, E. , Shahhosseini, Z. , Khani, S. , & Elyasi, F. (2023). Pain‐focused psychological interventions in women with endometriosis: A systematic review. Neuropsychopharmacology Reports, 43(3), 310–319. 10.1002/npr2.12348 37366616 PMC10496056

[bjhp12794-bib-0052] Schäfer, G. , Prkachin, K. M. , Kaseweter, K. A. , & Williams, A. C. (2016). Health care providers' judgments in chronic pain: The influence of gender and trustworthiness. Pain, 157(8), 1618–1625. 10.1097/j.pain.0000000000000536 26934512

[bjhp12794-bib-0053] Seear, K. (2009). ‘Nobody really knows what it is or how to treat it’: Why women with endometriosis do not comply with healthcare advice. Health, Risk & Society, 11(4), 367–385. 10.1080/13698570903013649

[bjhp12794-bib-0054] Seear, K. (2016). The makings of a modern epidemic: Endometriosis, gender and politics. Routledge. 10.4324/9781315555782

[bjhp12794-bib-0055] Sims, O. T. , Gupta, J. , Missmer, S. A. , & Aninye, I. O. (2021). Stigma and endometriosis: A brief overview and recommendations to improve psychosocial well‐being and diagnostic delay. International Journal of Environmental Research and Public Health, 18(15), 8210. 10.3390/ijerph18158210 34360501 PMC8346066

[bjhp12794-bib-0056] Singh, S. S. , Gude, K. , Perdeaux, E. , Gattrell, W. T. , & Becker, C. M. (2020). Surgical outcomes in patients with endometriosis: A systematic review. Journal of Obstetrics and Gynaecology Canada, 42(7), 881–888. 10.1016/j.jogc.2019.08.004 31718952

[bjhp12794-bib-0057] Todd, J. , Pickup, B. , & Coutts‐Bain, D. (2023). Fear of progression, imagery, interpretation bias, and their relationship with endometriosis pain. Pain, 164(12), 2839–2844. 10.1097/j.pain.0000000000003003 37530656

[bjhp12794-bib-0058] Turan, J. M. , Elafros, M. A. , Logie, C. H. , Banik, S. , Turan, B. , Crockett, K. B. , Pescosolido, B. , & Murray, S. M. (2019). Challenges and opportunities in examining and addressing intersectional stigma and health. BMC Medicine, 17(1), 7. 10.1186/s12916-018-1246-9 30764816 PMC6376691

[bjhp12794-bib-0059] Ugwumadu, L. , Chakrabarti, R. , Williams‐Brown, E. , Rendle, J. , Swift, I. , John, B. , Allen‐Coward, H. , & Ofuasia, E. (2017). The role of the multidisciplinary team in the management of deep infiltrating endometriosis. Gynecological Surgery, 14(1), 15. 10.1186/s10397-017-1018-0 28890677 PMC5570783

[bjhp12794-bib-0060] Van Niekerk, L. , Weaver‐Pirie, B. , & Matthewson, M. (2019). Psychological interventions for endometriosis‐related symptoms: A systematic review with narrative data synthesis. Archives of Women's Mental Health, 22(6), 723–735. 10.1007/s00737-019-00972-6 31081520

[bjhp12794-bib-0061] Vercellini, P. , Crosignani, P. G. , Abbiati, A. , Somigliana, E. , Viganò, P. , & Fedele, L. (2009). The effect of surgery for symptomatic endometriosis: The other side of the story. Human Reproduction Update, 15(2), 177–188. 10.1093/humupd/dmn062 19136455

[bjhp12794-bib-0062] Vercellini, P. , Fedele, L. , Aimi, G. , Pietropaolo, G. , Consonni, D. , & Crosignani, P. G. (2007). Association between endometriosis stage, lesion type, patient characteristics and severity of pelvic pain symptoms: A multivariate analysis of over 1000 patients. Human Reproduction, 22(1), 266–271. 10.1093/humrep/del339 16936305

[bjhp12794-bib-0063] Vercellini, P. M. D. , Barbara, G. M. D. , Somigliana, E. M. D. P. D. , Bianchi, S. M. D. , Abbiati, A. M. D. , & Fedele, L. M. D. (2010). Comparison of contraceptive ring and patch for the treatment of symptomatic endometriosis. Fertility and Sterility, 93(7), 2150–2161. 10.1016/j.fertnstert.2009.01.071 19328469

[bjhp12794-bib-0064] Viane, I. , Crombez, G. , Eccleston, C. , Poppe, C. , Devulder, J. , Van Houdenhove, B. , & De Corte, W. (2003). Acceptance of pain is an independent predictor of mental well‐being in patients with chronic pain: Empirical evidence and reappraisal. Pain, 106(1), 65–72. 10.1016/S0304-3959(03)00291-4 14581112

[bjhp12794-bib-0065] Vlaeyen, J. W. S. , & Linton, S. J. (2000). Fear‐avoidance and its consequences in chronic musculoskeletal pain: A state of the art. Pain, 85(3), 317–332. 10.1016/S0304-3959(99)00242-0 10781906

[bjhp12794-bib-0066] Woldhuis, T. , & Gandy, M. (2024). Illness invalidation and psychological distress in adults with chronic physical health symptoms. General Hospital Psychiatry, 91, 89–95. 10.1016/j.genhosppsych.2024.10.001 39426073

[bjhp12794-bib-0067] Young, K. , Fisher, J. , & Kirkman, M. (2015). Women's experiences of endometriosis: A systematic review and synthesis of qualitative research. The Journal of Family Planning and Reproductive Health Care, 41(3), 225–234. 10.1136/jfprhc-2013-100853 25183531

[bjhp12794-bib-0068] Young, K. , Fisher, J. , & Kirkman, M. (2017). Clinicians' perceptions of women's experiences of endometriosis and of psychosocial care for endometriosis. Australian and New Zealand Journal of Obstetrics and Gynaecology, 57(1), 87–92. 10.1111/ajo.12571 28251627

[bjhp12794-bib-0069] Young, K. , Fisher, J. , & Kirkman, M. (2019). “Do mad people get endo or does endo make you mad?”: Clinicians' discursive constructions of medicine and women with endometriosis. Feminism & Psychology, 29(3), 337–356. 10.1177/0959353518815704

[bjhp12794-bib-0070] Young, K. , Fisher, J. , & Kirkman, M. (2020). Partners instead of patients: Women negotiating power and knowledge within medical encounters for endometriosis. Feminism & Psychology, 30(1), 22–41. 10.1177/0959353519826170

[bjhp12794-bib-0071] Zhang, L. , Losin, E. A. R. , Ashar, Y. K. , Koban, L. , & Wager, T. D. (2021). Gender biases in estimation of others' pain. The Journal of Pain, 22(9), 1048–1059. 10.1016/j.jpain.2021.03.001 33684539 PMC8827218

