# Supplementary material for: Development of the COMPASS model of endometriosis: A COmprehensive model of pain encompassing agency, systemic factors and sense making
Source: Br J Health Psychol. 2025 Mar 18;30(2):e12794. doi: 10.1111/bjhp.12794 (PMC11914864; doi:10.1111/bjhp.12794)
Supplement: Supplementary file 1 — Table S1. Figure S1. [file BJHP-30-0-s001.docx]

**Supplementary Files**

**Supplementary Table S1.**

Results of content analysis of participants’ responses to the question “What is the largest challenge people with endometriosis currently face?” (N = 841)

| Code | Sub code | Responses |
| --- | --- | --- |
| Invalidation | General community | 277 |
|  | Healthcare workers | 141 |
|  | Workplace | 9 |
|  | Total | 428 |
| Lack of understanding | General community | 164 |
|  | Health care professionals | 95 |
|  | Lack of knowledge | 55 |
|  | Workplaces | 24 |
|  | Misinformation | 9 |
|  | Total | 347 |
| Diagnosis | General | 147 |
|  | Delay | 73 |
|  | Misdiagnosis | 22 |
|  | Invasive | 18 |
|  | Total | 259 |
| Lack of treatment options | General | 106 |
|  | Pain relief | 47 |
|  | Long term | 20 |
|  | Total | 173 |
| Difficulty accessing care | General | 25 |
|  | Specialists | 28 |
|  | Cost of care | 49 |
|  | Total | 102 |
| Pain and its impacts |  | 99 |
| Stigma | General | 39 |
|  | Gendered nature of condition and pain | 51 |
|  | Total | 90 |
| Workplace challenges | General | 44 |
|  | Attendance | 15 |
|  | Lack of sick leave | 14 |
|  | Total | 73 |
| Pain management | General | 77 |
|  | Lack of advice from medical professionals | 11 |
|  | Total | 68 |
| Invisible nature of condition |  | 52 |
| Lack of cure |  | 49 |
| Symptom burden |  | 45 |
| Impact of pain |  | 45 |
| Fertility impacts |  | 38 |
| Lack of support |  | 27 |
| Impact on mental health |  | 26 |
| Unpredictability and uncertainty |  | 23 |
| Lack of research and funding |  | 22 |
| Impact on relationships | General | 11 |
|  | Sexual functioning | 5 |
|  | Total | 16 |
| Communicating pain severity |  | 13 |
| Lack of integrated MDT care |  | 10 |

**Supplementary Table S2.**

Results of content analysis of participants’ responses to the question “Whilst a cure would be the best outcome, what could be done in the meantime to make a difference to people living with endometriosis?” (N = 808)

| Code | Sub code | Responses |
| --- | --- | --- |
| More education | General community | 81 |
|  | Doctors and medical staff | 158 |
|  | Workplaces | 23 |
|  | Teachers and schools | 24 |
|  | For people with endometriosis | 30 |
|  | Total | 325 |
| Greater understanding | General community | 68 |
|  | Greater awareness | 115 |
|  | Workplaces | 33 |
|  | Doctors and health professionals | 28 |
|  | Total | 244 |
| Better access to treatment and care | General | 26 |
|  | Specialists | 47 |
|  | Affordable treatments | 39 |
|  | Pain relief | 26 |
|  | Shorter wait times | 16 |
|  | Pain management | 10 |
|  | Total | 162 |
| Financial Support | General | 47 |
|  | Medicare subsidies | 39 |
|  | Disability support pension | 10 |
|  | More sick leave | 26 |
|  | Total | 122 |
| Validation | General | 119 |
| Support | Workplace | 27 |
|  | Mental health | 15 |
|  | Continuity of care | 6 |
|  | Endometriosis specific centers | 6 |
|  | For caregivers | 4 |
|  | Groups | 4 |
|  | Disability support | 3 |
|  | Following diagnosis | 2 |
|  | Total | 108 |
| Better treatment options | General | 47 |
|  | Alternative treatments | 15 |
|  | Non-invasive | 12 |
|  | Less side effects | 11 |
|  | Early intervention | 4 |
|  | More treatment options | 2 |
|  | Total | 91 |
| Pain management | General | 59 |
|  | Plans | 26 |
|  | More services | 2 |
|  | Total | 95 |
| Diagnosis | General | 8 |
|  | Faster | 42 |
|  | Non-invasive methods | 21 |
|  | Easier | 12 |
|  | Total | 82 |
| More research and funding |  | 67 |
| Pain relief | General | 23 |
|  | Accessible | 25 |
|  | Non-opioid | 7 |
|  | Total | 56 |
| MDT care |  | 53 |
| More recognition of the condition |  | 31 |
| Reducing stigma |  | 27 |
| Consistent messaging across health professionals |  | 8 |

**Supplementary Table S3.**

Results of content analysis of participants’ responses to the question “Whilst waiting for a cure, what could be done to improve pain management for people living with endometriosis?” (N = 735)

| Code | Sub code | Responses |
| --- | --- | --- |
| Better access to treatment and care | General | 54 |
|  | Pain relief | 118 |
|  | MDT care | 38 |
|  | Affordable treatment | 32 |
|  | Specialists | 24 |
|  | Total | 256 |
| MDT care | General/Total | 97 |
| Better treatments | General | 16 |
|  | Pain relief | 43 |
|  | Less side effects/noninvasive | 18 |
|  | Alternative treatments | 9 |
|  | More options | 6 |
|  | Total | 92 |
| Financial support | General | 38 |
|  | Subsidised care (medical and allied health) | 33 |
|  | More sick leave | 9 |
|  | Total | 80 |
| More education | General | 15 |
|  | For healthcare professionals | 57 |
|  | Total | 72 |
| Pain management plans | General/Total | 56 |
| More pain management information and self-management resources | General/Total | 54 |
| More support | General | 15 |
|  | Flexible working arrangements | 16 |
|  | Treatment | 16 |
|  | Mental health | 5 |
|  | Total | 50 |
| Validation | General/total | 50 |
| Reducing stigma | General | 7 |
|  | Around pain medication | 31 |
|  | Total | 38 |
| More research into effective pain management and treatments | General/Total | 37 |
| More awareness | General | 22 |
|  | Workplaces | 9 |
|  | Total | 31 |
| More understanding | General | 7 |
|  | Healthcare professionals | 16 |
|  | Total | 23 |
| Faster diagnosis | General/total | 5 |

**Supplementary Table S4.** Comparing pain outcomes between those who did and did not leave qualitative responses.

|  | Left response | | Did not leave response | |  |  |  |
| --- | --- | --- | --- | --- | --- | --- | --- |
|  | M | SD | M | SD | t | df | p |
| Pain severity | 7.37 | 2.33 | 7.79 | 2.69 | -1.031 | 873 | .303 |
| Pain distress | 5.89 | 2.23 | 6.29 | 2.69 | -1.00 | 873 | .317 |
| Pain impact | 17.10 | 6.11 | 18.65 | 6.34 | -1.40 | 873 | .148 |

Note: N = 841 left response, N = 34 did not.

**Supplementary Figure S1.** The COMPASS model representing the relationships between themes of endometriosis-related pain experiences associated with elevated pain-related distress and impact. Additional systemic factors identified through content analysis are depicted by the dashed line box.

Figure S1 is the inverse presentation of the model which proposes that the systemic gendered nature of pain hinders awareness and knowledge about endometriosis and gives rise to invalidation. Invalidation can be experienced through unhelpful interactions with healthcare professionals and family, as well as diagnosis delays, and leads people to distrust healthcare system. Continued experiences of invalidation perpetuate this distrust, hinder one’s sense of agency and lead people to try to make sense of their condition, such as identifying with endometriosis and pain. Together, these processes culminate in exacerbating the burden of endometriosis and pain related distress and impact.

**
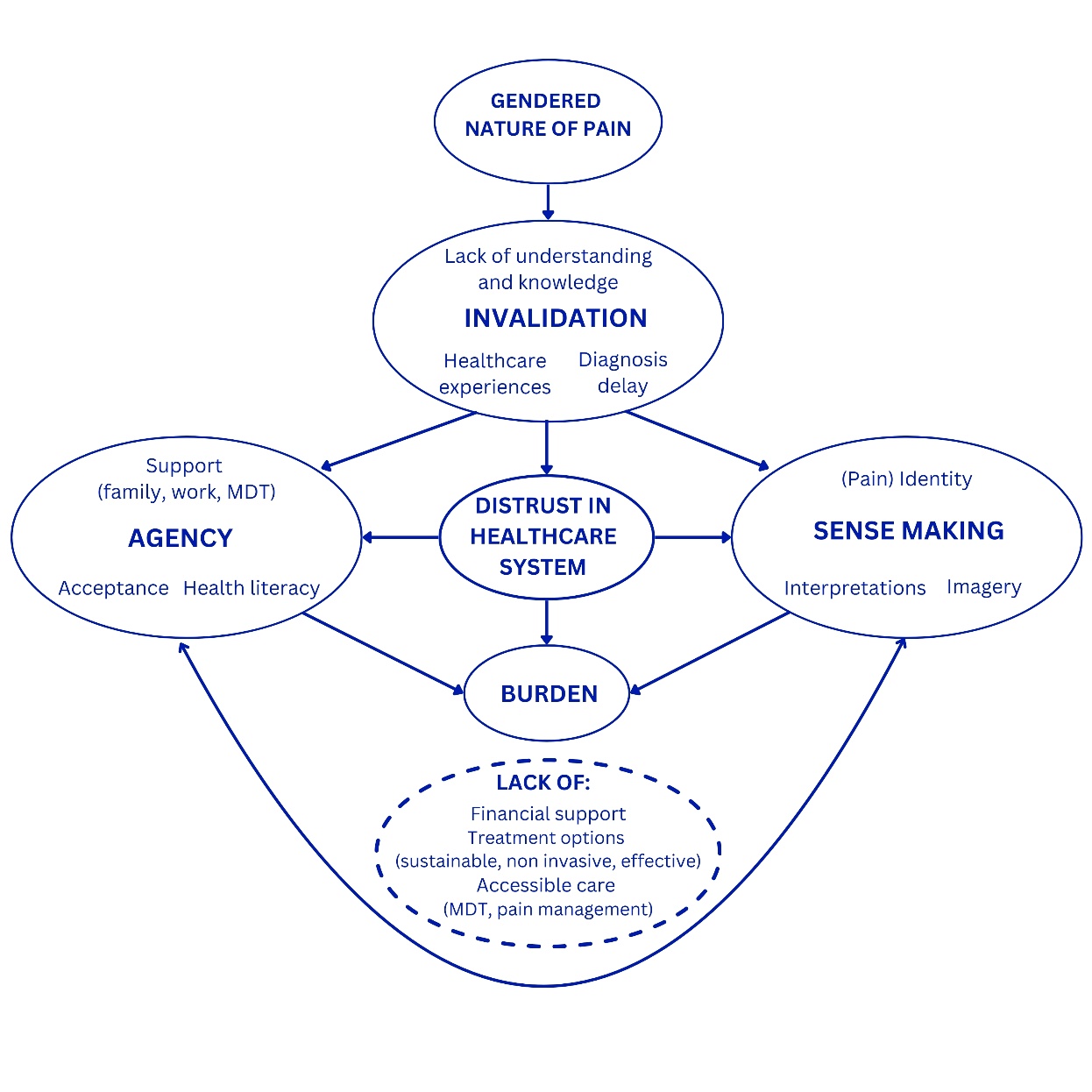
**
